# Spatiotemporal variations in urban CO_2_ flux with land-use types in Seoul

**DOI:** 10.1186/s13021-022-00206-w

**Published:** 2022-05-03

**Authors:** Chaerin Park, Sujong Jeong, Moon-Soo Park, Hoonyoung Park, Jeongmin Yun, Sang-Sam Lee, Sung-Hwa Park

**Affiliations:** 1 Department of Environmental Planning, Graduate School of Environmental Studies, Seoul National University, Seoul, Republic of Korea; 2 Department of Climate and Environment, Sejong University, Seoul, Republic of Korea; 3 National Institute of Meteorological Sciences, 63568 Jeju, Republic of Korea

**Keywords:** CO_2_ flux, Annual cycle of CO_2_ flux, Diurnal cycle of CO_2_ flux, Urban, Eddy covariance, CO_2_ emissions, Land-use types, Seoul

## Abstract

**Background:**

Cities are a major source of atmospheric CO_2_; however, understanding the surface CO_2_ exchange processes that determine the net CO_2_ flux emitted from each city is challenging owing to the high heterogeneity of urban land use. Therefore, this study investigates the spatiotemporal variations of urban CO_2_ flux over the Seoul Capital Area, South Korea from 2017 to 2018, using CO_2_ flux measurements at nine sites with different urban land-use types (baseline, residential, old town residential, commercial, and vegetation areas).

**Results:**

Annual CO_2_ flux significantly varied from 1.09 kg C m^− 2^ year^− 1^ at the baseline site to 16.28 kg C m^− 2^ year^− 1^ at the old town residential site in the Seoul Capital Area. Monthly CO_2_ flux variations were closely correlated with the vegetation activity (r = − 0.61) at all sites; however, its correlation with building energy usage differed for each land-use type (r = 0.72 at residential sites and r = 0.34 at commercial sites). Diurnal CO_2_ flux variations were mostly correlated with traffic volume at all sites (r = 0.8); however, its correlation with the floating population was the opposite at residential (r = − 0.44) and commercial (r = 0.80) sites. Additionally, the hourly CO_2_ flux was highly related to temperature. At the vegetation site, as the temperature exceeded 24 ℃, the sensitivity of CO_2_ absorption to temperature increased 7.44-fold than that at the previous temperature. Conversely, the CO_2_ flux of non-vegetation sites increased when the temperature was less than or exceeded the 18 ℃ baseline, being three-times more sensitive to cold temperatures than hot ones. On average, non-vegetation urban sites emitted 0.45 g C m^− 2^ h^− 1^ of CO_2_ throughout the year, regardless of the temperature.

**Conclusions:**

Our results demonstrated that most urban areas acted as CO_2_ emission sources in all time zones; however, the CO_2_ flux characteristics varied extensively based on urban land-use types, even within cities. Therefore, multiple observations from various land-use types are essential for identifying the comprehensive CO_2_ cycle of each city to develop effective urban CO_2_ reduction policies.

## Background

The global atmospheric CO_2_ concentration has exceeded 400 ppm since 2013 because of the increasing use of fossil fuels [1]. Currently, more than 70% of the CO_2_ emissions from fossil fuel combustion occur in cities [2, 3]. Although cities are major sources of atmospheric CO_2_, they also present a major opportunity to reduce atmospheric CO_2_. The net CO_2_ flux from cities results from the combined effects of anthropogenic emissions and vegetation uptake [4]. Therefore, understanding the comprehensive surface CO_2_ exchange processes, including CO_2_ sources and sinks, is a prerequisite for developing effective policies to reduce net CO_2_ flux from cities.

Traditionally, inventory data that estimate CO_2_ emissions based on fossil fuel consumption statistics are utilized to understand variations in CO_2_ flux emitted from cities worldwide [5–7]. Recently developed sophisticated inventory data have facilitated the identification of CO_2_ emission characteristics at the building and road scales according to each emission sector [7]. However, these spatiotemporally high-resolution inventory data were only established for a limited number of cities. For most cities, aggregated inventory data at the municipal scale cannot fully reflect the spatiotemporal heterogeneity of CO_2_ characteristics. Moreover, the current state of data will not be available until at least one or two years from now [1, 8]. Additionally, there are substantial uncertainties in the city-level inventories because of various emission sources and insufficient statistical data within cities [9, 10]. Gurney et al. [11] suggested that 48 cities in the United States under-reported their emissions by 18.3%. This means that although inventory data enables quantification of urban CO_2_ emissions, solely using this data poses limitations on the appropriate management of urban CO_2_ emissions.

To supplement the inventory data and improve the understanding of real-time urban CO_2_ variability, CO_2_ observation networks have been established in many cities; studies based on these observations have been conducted actively [12–21]. Among them, CO_2_ flux observations through eddy covariance (EC) directly measure the net CO_2_ exchange rate per unit area [22]. Initially, EC observations were used to measure the CO_2_ flux in a vegetated area [23]. EC observations over urban areas have increased with the increasing importance of understanding urban net CO_2_ flux [12, 13, 15, 17, 20, 24–27]. EC observations in cities provide various information on the complex CO_2_ exchange processes as the EC-based CO_2_ flux represents the integrated response of CO_2_ to anthropogenic emissions, vegetation uptake, and meteorological conditions [22, 28].

Using the characteristics of CO_2_ flux data, previous studies quantified the net CO_2_ flux in multiple cities and analyzed the regional heterogeneity of the CO_2_ flux. Stagakis et al. [20] quantified the annual CO_2_ flux in Heraklion, Greece as 5.29 kg C m^− 2^ year^− 1^ and determined the contribution of multiple sources (i.e., vehicle, heating, and human respiration) to CO_2_ flux. CO_2_ flux observations in London, Helsinki, Beijing, and Vancouver showed that they emit 12.72, 1.76, 4.90, and 6.71 kg C m^− 2^ year^− 1^ of CO_2_ per year, respectively, serving as significant CO_2_ sources [12, 13, 15, 24]. Moreover, Ueyama and Ando [17] found that the CO_2_ flux varies greatly, from 0.5 to 4.9 kg C m^− 2^ year^− 1^, in Sakai (Japan) depending on the degree of urbanization. According to the CO_2_ flux measurements in Seoul, South Korea, residential areas emit 40 times more CO_2_ than urban park areas during the non-growing season (March) [25]. Accordingly, recent studies have shown different CO_2_ flux characteristics within and between cities because of various land-use types. Therefore, more urban EC measurements need to be conducted [29]. Additionally, most previous studies used a limited number of observation sites (one to three); consequently, understanding the spatiotemporal characteristics of the CO_2_ flux according to various land-use types in a city remains challenging.

Therefore, this study investigated the EC-based CO_2_ flux data measured at nine sites with different urban land-use types around the Seoul Capital Area, South Korea, the world’s largest carbon-producing urban agglomeration, and aimed to improve the understanding of the CO_2_ cycle in urban areas with various surface characteristics [21, 30–33]. The specific objectives of this study were to: (1) quantify the CO_2_ flux over the Seoul Capital Area considering different urban land-use types (baseline, residential, old town residential, commercial, and vegetation areas), (2) understand the characteristics of temporal CO_2_ flux variations for each surface type, and (3) evaluate the driving factors of CO_2_ flux variations.

## Methods

### Nine sites around the Seoul Capital Area

Seoul, South Korea, one of the largest megacities in East Asia, is a densely populated city, with approximately 25 million residents in the Seoul Capital Area, more than 50% of the total population of the country [34]. Seoul has a temperate climate, with a minimum monthly mean temperature of − 2.4 ℃ in January and a maximum monthly mean temperature of 25.7 ℃ in August (data from 1981 to 2010) according to the Korea Meteorological Administration (KMA) [35]. The annual mean precipitation is 1450.5 mm, with more than 60% occurring in the summer (June, July, and August).

**Fig. 1:**
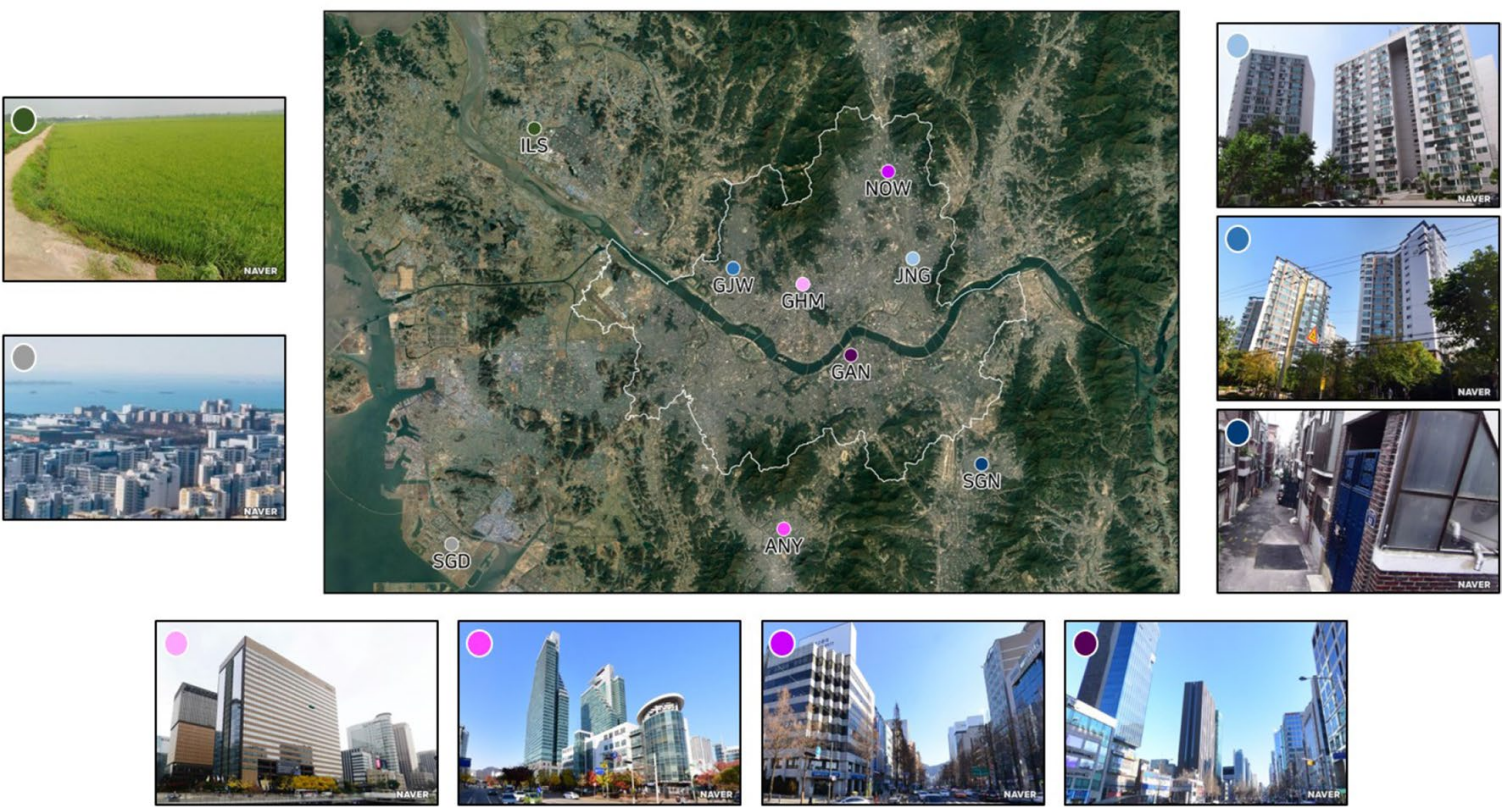
Location of CO_2_ flux observation sites in the Seoul Capital Area with images representing each observation site. The images were provided by NAVER Corp

In this study, measurements at nine sites around the Seoul Capital Area were used to study the spatiotemporal variations in the urban CO_2_ flux [36]. Each observation site was located at a place representative of different urban land-use types based on the surrounding topography and geography (Table 1; Fig. 1). Among the nine sites, Songdo (SGD; 37.383° N, 126.655° E) was used as the baseline site. This site is located at the end of the west coast of South Korea and has minimal impact on CO_2_ emissions from the Seoul Capital Area. By selecting SGD, which is also characterized by reduced vegetation and human activities, as a baseline site, we attempted to contrast variation of CO_2_ flux at urban sites characterized by considerable vegetation and intensive residential and commercial activities with those of areas that are not.

The other sites were broadly classified into three land-use types: residential, commercial, and vegetation areas. For land-use type determination, we used a 1 m spatial resolution land-cover map created by the Ministry of Environment [37]. The land-use type of each site was defined as the most dominant land-use type, except for roads and urban vegetation, within a 1 km radius of the observation site. Jungnang (JNG; 37.591° N, 127.079° E), Gajwa (GJW; 37.584° N, 126.914° E), and Seongnam (SGN; 37.441° N, 127.142° E) were classified as residential areas. Unlike JNG and GJW, SGN consists of low-rise old town residential areas with low combustion efficiency. Among all buildings in SGN, only 7% of them were built after 2000 according to the GIS building integrated information provided by the Ministry of Land, Infrastructure and Transport [38]. In contrast, in JNG and GJW, more than 23% of all buildings were constructed after 2000. Gwanghwamun (GHM; 37.572° N, 126.978° E), Anyang (ANY; 37.394° N, 126.960° E), Nowon (NOW; 37.654° N, 127.056° E), and Gangnam (GAN; 37.520° N, 127.022° E) were classified as commercial areas. A land-cover map for Ilsan (ILS; 37.685° N, 126.731° E) was unavailable as it is located near a military base. However, as more than 70% of the area within a 1 km radius of ILS is farmland according to satellite imagery, we defined ILS as a vegetation area (Fig. 1).

**Fig. 2:**
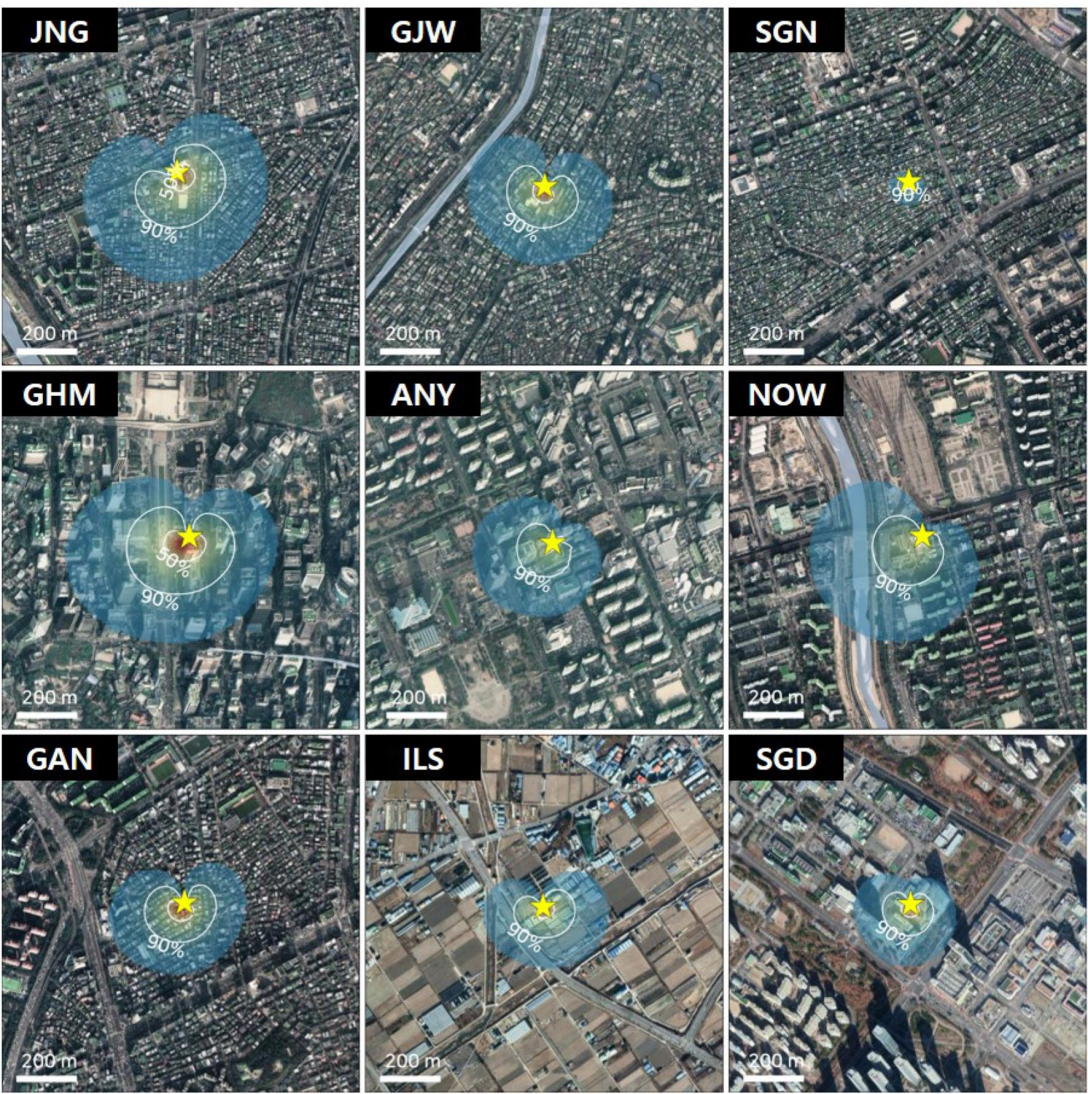
Aerial images of 1.5 × 1.5 km area of each site (star), with the average footprint area for the study period (January 2017 to December 2018)

The footprint of each site was calculated using the analytical footprint model from Kormann and Meixer [39]; the average footprint of each site was calculated by averaging the daily mean footprints for the entire study period (January 2017 to December 2018; Fig. 2). The model indicates that the majority of the flux at each site originated within 200–500 m of the EC tower. The footprint area affecting each site consisted of regions representing the land-use type of each site. Although there were differences in the footprint areas of each site, the surrounding land-use types were homogeneously distributed; this meant that CO_2_ flux observations represented each land-use type irrespective of the footprint area.

### Instrumentation and data processing instrumentation and data processing

Fourteen EC observation systems have been operated by the National Institute of Meteorological Sciences (NIMS) since 2013. The EC tower observation system is equipped with an open path gas analyzer (EC150, Campbell Scientific Inc.) and sonic anemometer (CSAT3A, Campbell Scientific Inc.). The open path gas analyzer, which measures atmospheric CO_2_ concentrations and water vapor, is calibrated every 6 months. The sonic anemometer directly measures three orthogonal wind components and the speed of sound. The EC towers are installed on the roof of each observation site (Table 1). Observations were made at a temporal resolution of 10 Hz for each EC tower; the 10 Hz raw data were pre-processed to 30 min-averaged flux data after four key procedures were conducted by NIMS: (1) verification of the physical limit; (2) removal of spike data [40]; (3) calculation of 30 min-averaged vertical flux [41]; and (4) Webb-Pearman-Leuning correction [42, 43]. Each EC tower observation system is built with integrated meteorological sensors to observe fundamental weather variables including temperature and precipitation. For more information on the observation system and data pre-processing, refer to Park et al. [36].

**Fig. 3:**
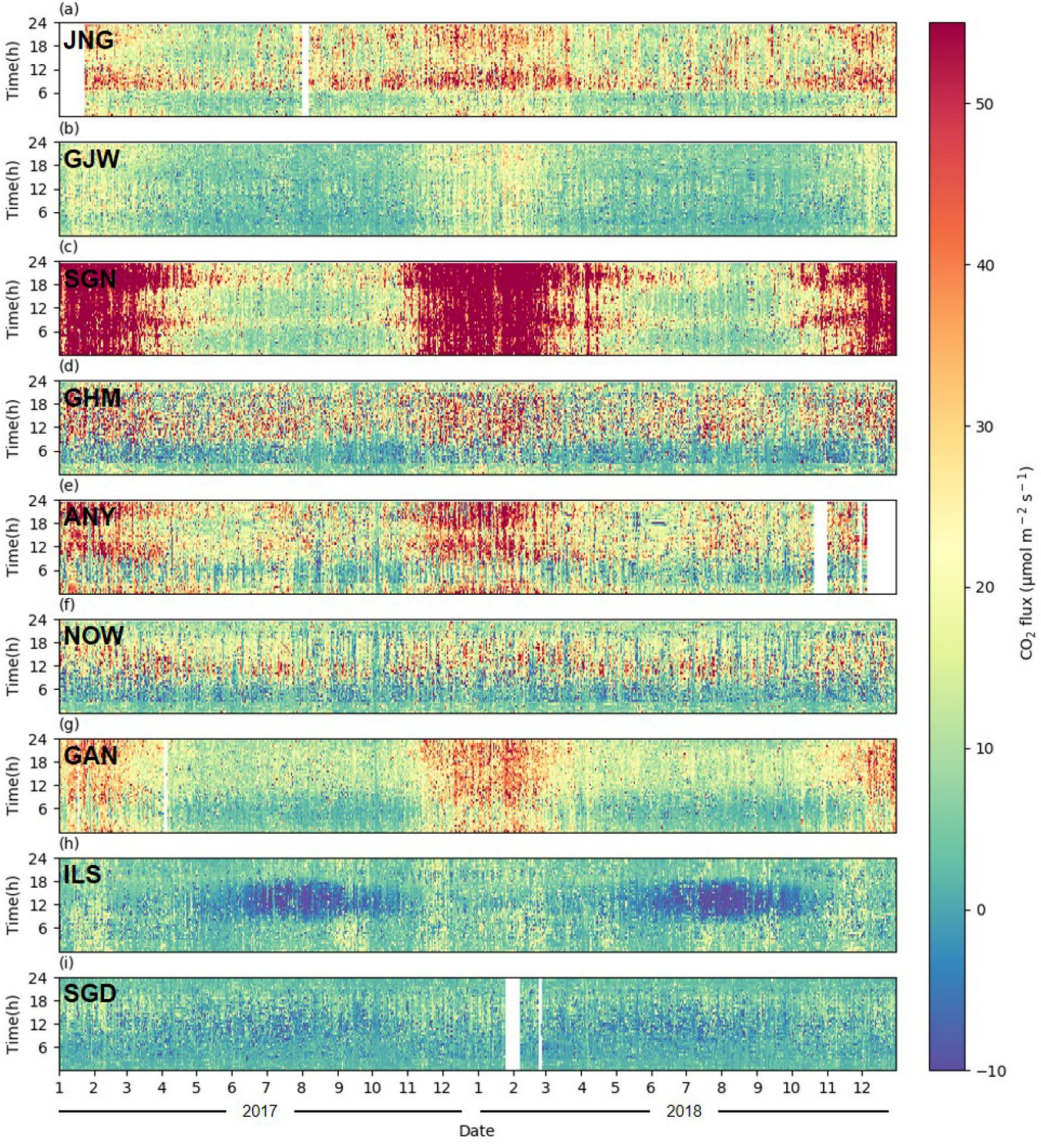
Hovmöller diagram showing the variations in CO_2_ flux by day (x-axis) and time of day (y-axis) at the nine sites based on half-hourly observation data from 2017 to 2018

In this study, two-year data were used from January 2017 to December 2018, when continuous observations were recorded at all sites. For the study period, we filtered the data collected at the 14 EC observation sites using half-hourly CO_2_ flux data pre-processed by the NIMS. Data filtering was conducted sequentially as follows: (1) CO_2_ flux data measured when the CO_2_ concentration was lower than the 1st percentile of that at the SGD baseline site were removed as the local urban effect of the observation site could not be well captured; (2) CO_2_ flux data measured during precipitation events and when the instrument malfunctioned were removed manually; (3) negative CO_2_ flux values measured at night (21:00–03:00) were removed as they were considered to be erroneous; and (4) major outliers were statistically filtered using the interquartile range method [44]. Here, major outliers considerably deviating from other values were removed as they were considered to poorly represent the surrounding environment of each site. Among the 14 EC observation sites, only the 9 sites with more than 80% data remaining after filtering were considered for analysis. The data coverage of the selected sites was 86.80% during the study period. For values with a period of missing data of less than 2 days, gap-filling was conducted in two stages: (1) if the missing period was shorter than 2 h, the missing value was reconstructed by linear interpolation of the values 1 h before and after the missing time; (2) if the missing period was more than 2 h and less than 2 days, the missing value was reconstructed using the mean diurnal variation of 3 days before and after the observation date. Gap-filling was not conducted for periods with data missing for longer than 2 days. After gap-filling, the EC data coverage at each observation site was at least 94%. Full data coverage (100% data availability) was achieved at all sites except at JNG (96.03%), ANY (94.25%), GAN (99.18%), and SGD (97.82%; Fig. 3).

### Ancillary data

To examine how each factor affected the monthly CO_2_ flux variability, normalized difference vegetation index (NDVI) and building gas-usage data were studied. Floating population, traffic volume, and temperature data were used to determine the factors causing diurnal variations in the CO_2_ flux.

We used the NDVI data from the Moderate Resolution Imaging Spectroradiometer (MODIS) sensor onboard the Terra satellite to assess the monthly correlation between the CO_2_ flux and vegetation activity [45]. Level 3 MODIS Vegetation Indices (MOD13Q1) Version 6 generates NDVI data every 16 days at a grid resolution of 250 m. We obtained the NDVI grid values within a 1 km radius from each CO_2_ flux site. The extracted average monthly NDVI values for each site were analyzed. The SGD site is located on coastal reclaimed land; therefore, the NDVI data were not calculated. Along with the NDVI data, building gas-usage, which is one of the largest sources of direct CO_2_ emissions over Seoul, was used to represent human activity [46]. Monthly building gas-usage data were obtained from the Ministry of Land, Infrastructure and Transport and can be downloaded from the Architectural Data System [47]. We utilized the total gas usage of all buildings except power plants in the administrative districts of each CO_2_ flux site during the study period.

Hourly floating population and traffic volume data are only observed within the administrative district of Seoul by the Seoul Metropolitan Government [48, 49]. Thus, we only used data for the JNG, GJW, GHM, NOW, and GAN sites, which are located in the administrative district of Seoul. The floating population data were calculated using the hourly mobile-phone LTE signals from certain points [50]. The total floating population data within the administrative district of each CO_2_ flux site were used. Traffic volume data were considered as inflows and outflows of vehicles for certain road sections. As the traffic volume is constant on a monthly basis, traffic volume data were only utilized for the analysis of diurnal variations in the CO_2_ flux. We used the traffic volume data measured at the closest point from each site. Observation points for the traffic volume of each site, located at places where the average traffic flow of nearby areas can be well captured, were 450 m to 2.4 km away from each CO_2_ flux site.

CO_2_ emission data for each site calculated from the inventory data were utilized to compare the observation-based CO_2_ flux and inventory-based CO_2_ emission datasets. Due to the absence of high spatiotemporal resolution CO_2_ inventory data for Seoul, the Open-Data Inventory for Anthropogenic Carbon dioxide (ODIAC) data was used. This dataset contains the highest resolution data available for Seoul, provided by the National Institute for Environmental Studies (NIES) in Japan [51]. This study used the latest version of ODIAC (ODIAC2020b), which provides monthly total fossil fuel CO_2_ emissions data with a spatial resolution of 1 km [52]; CO_2_ emission data closest to each observation site were utilized.

## Results

The Hovmöller diagram of the CO_2_ flux by day (x-axis) and time of day (y-axis) at the nine sites shows distinct differences in the temporal CO_2_ flux variations at each site in the Seoul Capital Area according to the land-use type (Fig. 3). Although there were clear seasonal and diurnal variations in the CO_2_ flux across all sites, their amplitudes and periods varied for each site. We further analyzed the spatiotemporal variations of the CO_2_ flux, as described in the following sections.

### Spatial variations

**Fig. 4:**
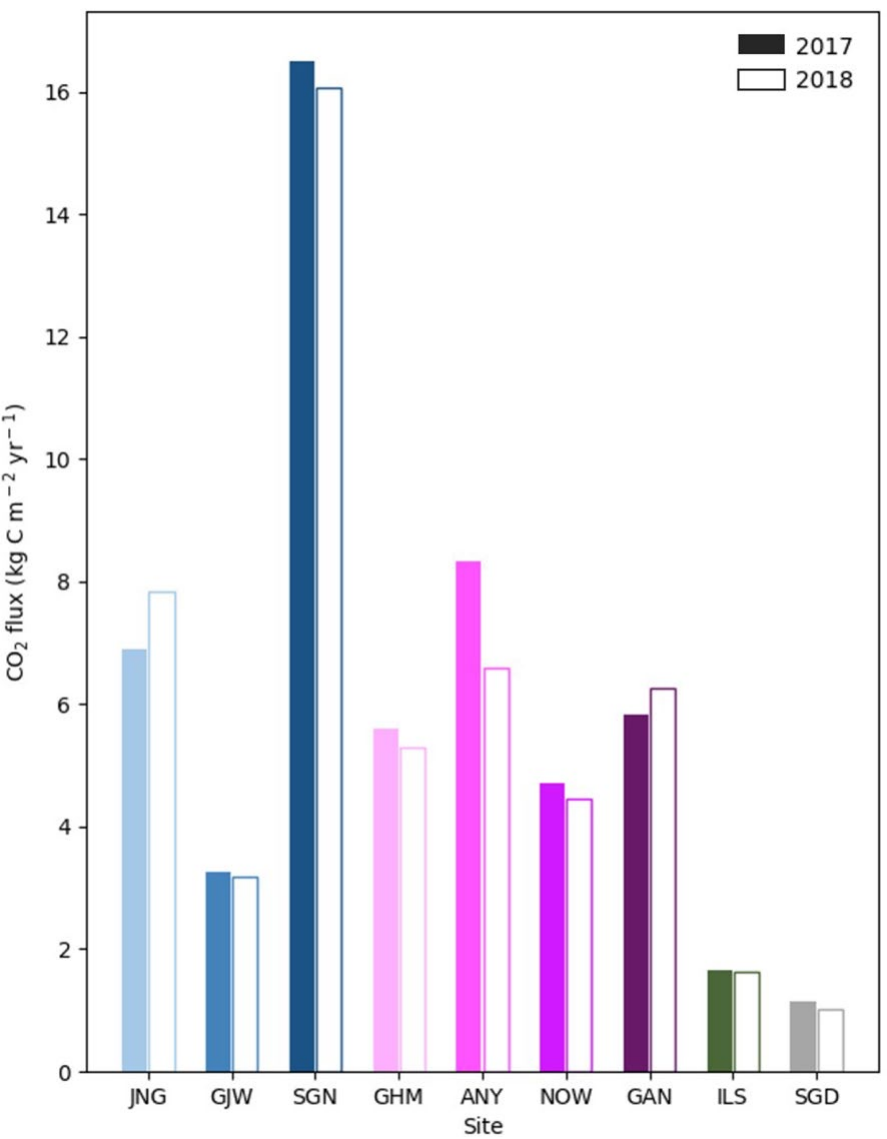
Bar graph of the annual total CO_2_ flux of each site. The colored bar represents the total annual CO_2_ flux in 2017 and the empty bar represents that in 2018

The average annual CO_2_ fluxes of each site in 2017 and 2018 significantly varied from 1.09 to 16.28 kg C m^− 2^ year^− 1^ according to the urban land-use type in the city. The average annual CO_2_ fluxes were the lowest in SGD (baseline site; 1.09 kg C m^− 2^ year^− 1^) and ILS (vegetation site; 1.65 kg C m^− 2^ year^− 1^; Fig. 4). Residential sites JNG, GJW, and SGN showed annual CO_2_ fluxes of 7.38, 3.23, and 16.28 kg C m^− 2^ year^− 1^, respectively. The commercial sites GHM, ANY, NOW, and GAN showed annual CO_2_ fluxes of 5.45, 7.47, 4.59, and 6.04 kg C m^− 2^ year^− 1^, respectively. On average, the CO_2_ flux of residential and commercial sites (7.20 kg C m^− 2^ year^− 1^) was seven times that of the baseline site and four times that of the vegetation site. In particular, the CO_2_ flux of the old town residential site (SGN) was three times that of other residential sites (5.30 kg C m^− 2^ year^− 1^). Although the amount of emitted CO_2_ differed for each site, all sites, including the vegetation and baseline sites, served as CO_2_ sources.

**Fig. 5:**
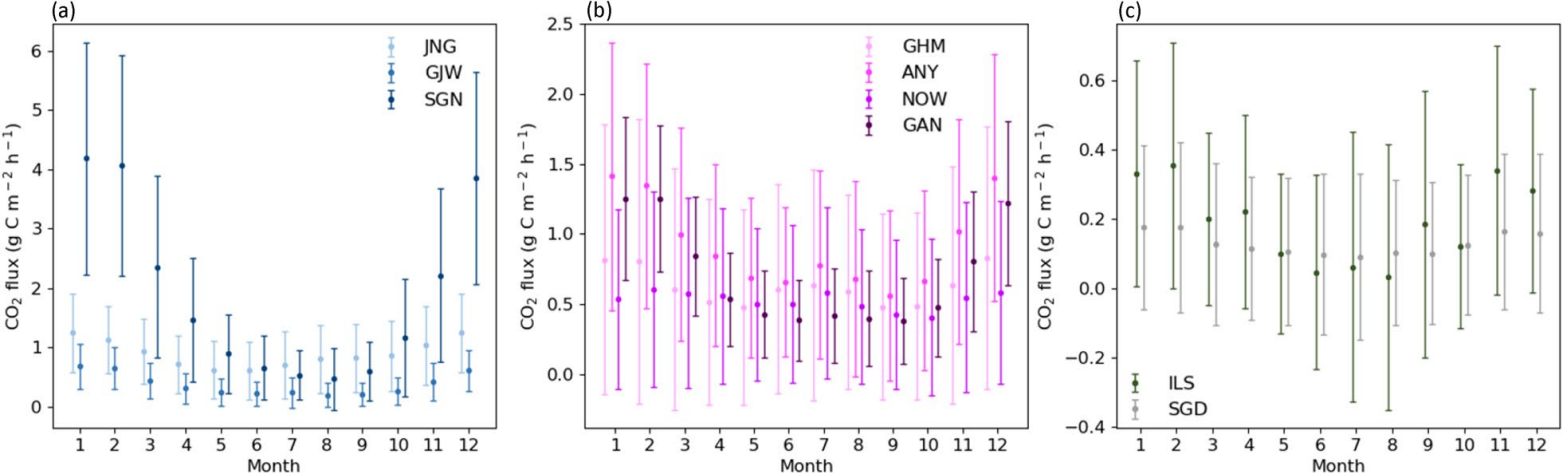
Time series of the monthly CO_2_ flux averaged for two years (2017 and 2018): **a** residential area, **b** commercial area, and **c** vegetation and baseline areas. Error bars indicate one standard deviation (±σ) of the mean CO_2_ flux of each month

### Monthly variations

All sites had clear seasonal variations with the lowest CO_2_ flux in the summer and the highest CO_2_ flux in the winter (Fig. 5). In general, the average CO_2_ flux for all sites was the lowest in August (0.42 g C m^− 2^ h^− 1^) and the highest in January (1.18 g C m^− 2^ h^− 1^). In summer, all sites released three times lower CO_2_ than in winter; however, even in summer, all sites, including the ILS vegetation site, had a positive CO_2_ flux. This indicates that all urban land-use types act as CO_2_ sources throughout the year. The highest seasonal amplitude (3.72 g C m^− 2^ h^− 1^), which is the difference between the monthly minimum and maximum CO_2_ fluxes, was observed at the SGN site. SGN emitted nine times more CO_2_ in winter than that in summer. The lowest seasonal amplitude was observed at the SGD baseline site (0.084 g C m^− 2^ h^− 1^). SGD released a relatively constant level of CO_2_ throughout the year compared to other sites.

**Fig. 6:**
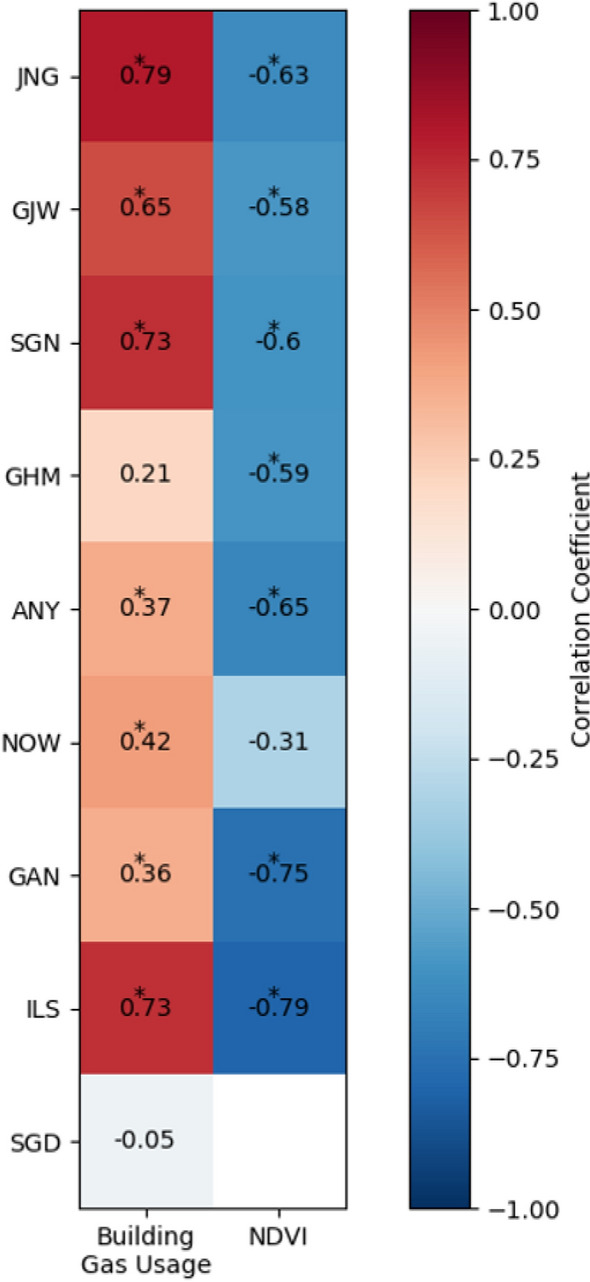
Heat map of the correlation coefficient matrix between the monthly CO_2_ flux and variables (building energy usage and normalized difference vegetation index (NDVI)) for each site from 2017 to 2018. The symbol * represents p < 0.1

**Fig. 7:**
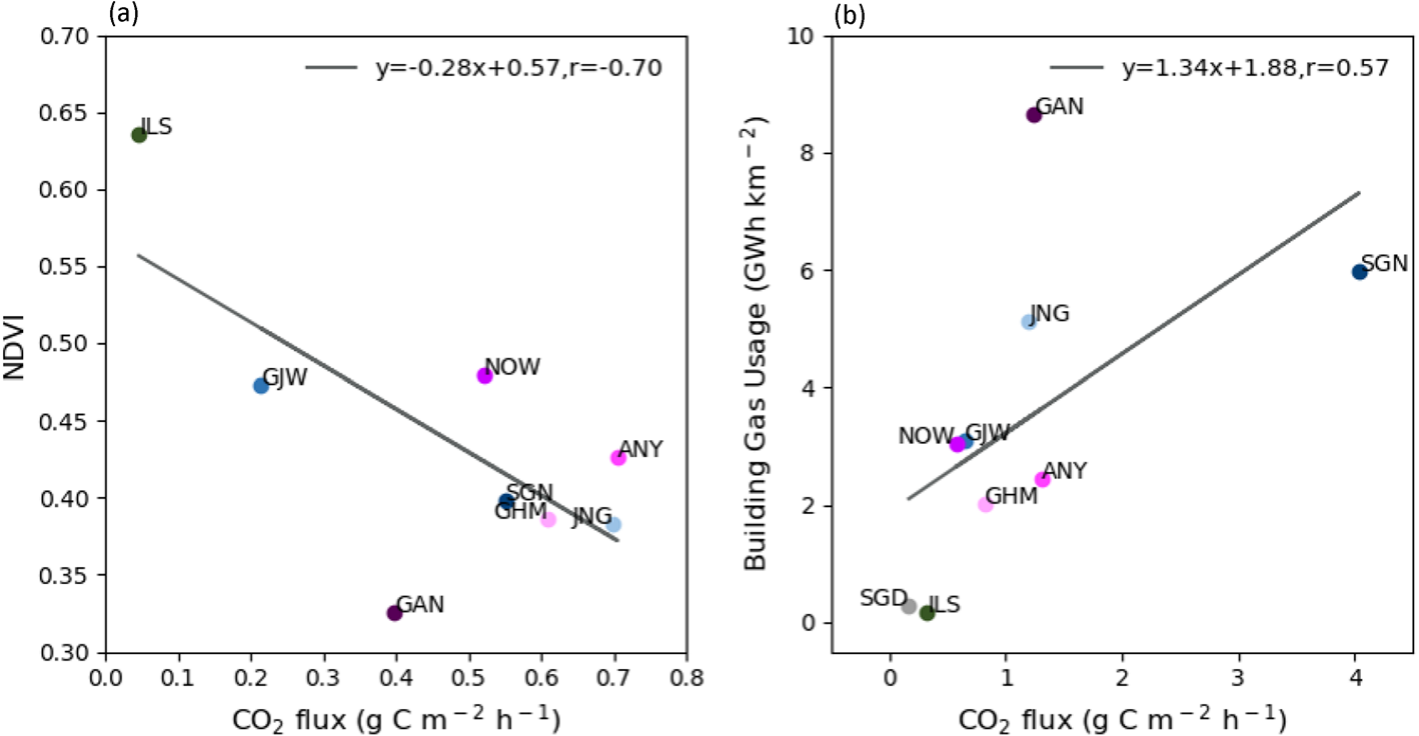
**a** Scatter plot of the monthly CO_2_ flux averaged during summer in accordance with the average monthly NDVI value during summer for each site. The solid line represents the linear regression between the CO_2_ flux and NDVI. **b** Scatter plot of the monthly CO_2_ flux averaged during winter in accordance with the average monthly building gas usage during winter for each site. The solid line represents the linear regression between the CO_2_ flux and building gas usage

To identify potential factors affecting monthly CO_2_ flux variations, relationships between CO_2_ flux and factors representing human activities and vegetation were assessed (Fig. 6). As traffic in the Seoul Capital Area does not substantially vary over the year, building energy usage, accounting for more than 70% of fossil fuel usage in Seoul, was used as the human activity factor [46]. Along with the human activity factor, the NDVI value represented the vegetation photosynthesis capacity and efficiency [53]. The monthly CO_2_ flux of all land-use types was positively correlated with the building gas usage and negatively correlated with NDVI data (Fig. 6). The correlation coefficient between the CO_2_ flux and building gas usage was relatively high in residential areas (r = 0.72, *p* < 0.1) and low in commercial areas (r = 0.34, *p* < 0.1). This was because of the different patterns of building gas usage in residential and commercial areas. In commercial areas, the maximum building gas usage in winter was similar to or less than that in summer. However, in residential areas, the maximum building gas usage in winter was considerably greater than that in summer (data not shown). On average, the NDVI values and CO_2_ flux showed a correlation coefficient of 0.61 at all sites, with the highest correlation coefficient of 0.79 at the ILS vegetation site. To further examine the effect of vegetation and building gas usage on the monthly variations in the CO_2_ flux, the relationship of the CO_2_ flux in each season with NDVI and building gas usage were analyzed (Fig. 7). In summer, sites with high NDVI values showed low CO_2_ flux values (r = − 0.70; Fig. 7a). In winter, sites with high building gas usage presented high CO_2_ flux values (r = 0.57; Fig. 7b). In particular, the CO_2_ absorption and emission by vegetation in summer and building gas usage in winter, respectively, play a vital role in the distinct monthly CO_2_ flux variations. The GAN site relatively deviated from the general trends of other sites as this was located near a river and was affected by strong atmospheric mixing.

**Fig. 8:**
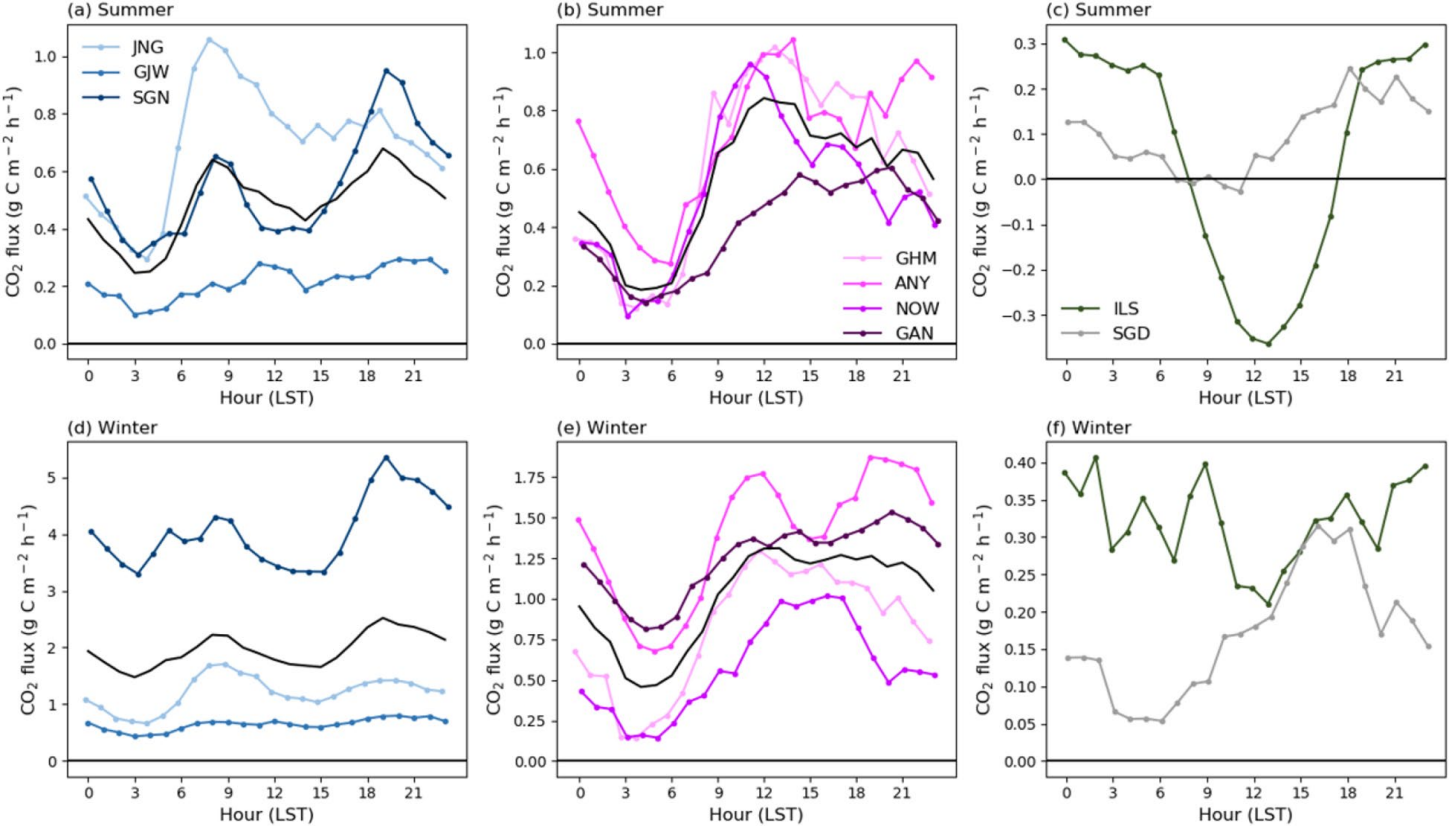
Diurnal time series of the CO_2_ flux using mean values of two years (2017 and 2018): **a** and **d** for residential, **b** and **e** commercial, and **c** and **f** vegetation and baseline sites **a**–**c** during summer and **d**–**f** winter. The black solid lines in **a**, **b**, **d**, and e represent the CO_2_ flux averaged for each land-use type

**Fig. 9:**
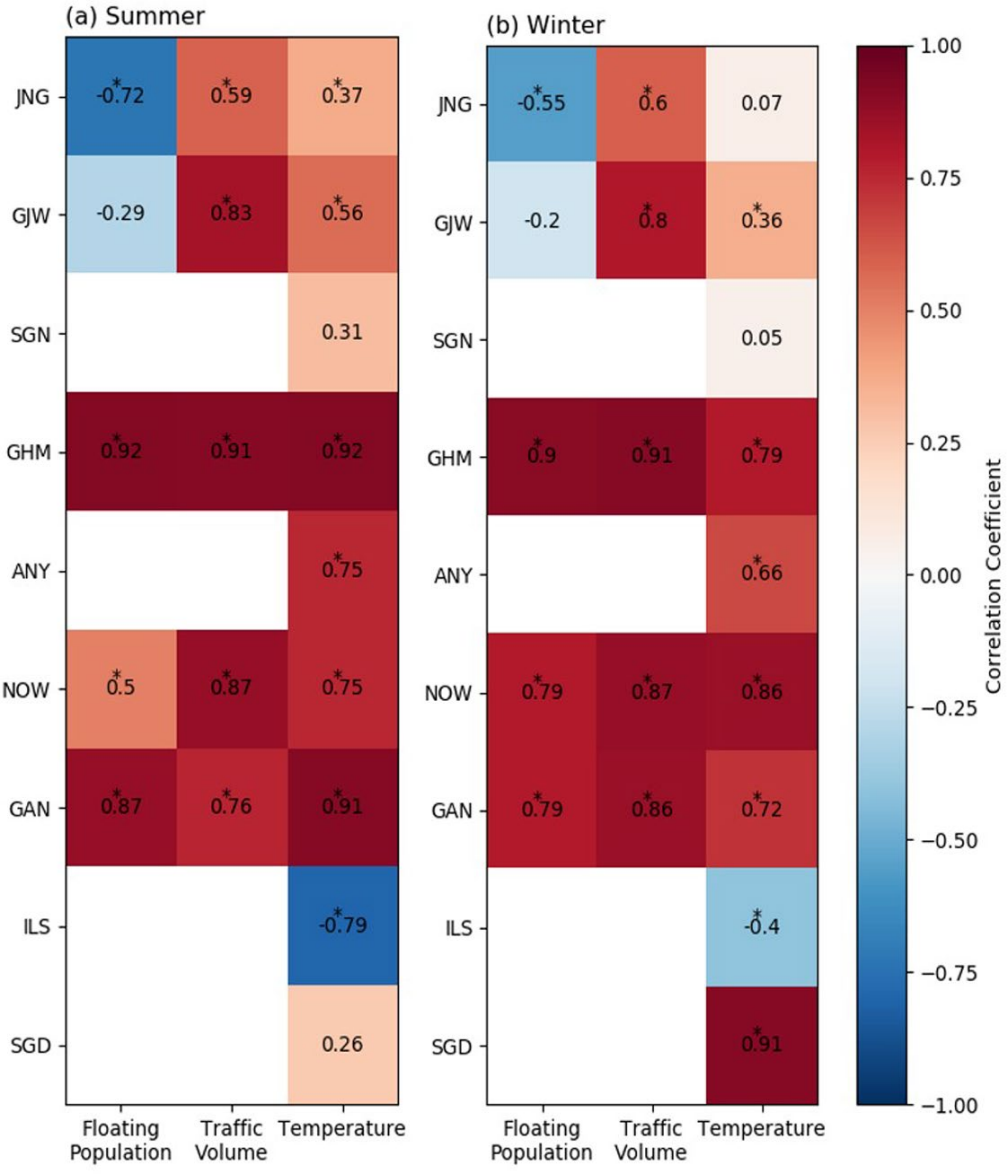
Heat map of the correlation coefficient matrix between the diurnal CO_2_ flux and variables (floating population, traffic volume, and temperature) for each site during **a** summer and **b** winter from 2017 to 2018. The symbol * represents p < 0.1

**Fig. 10:**
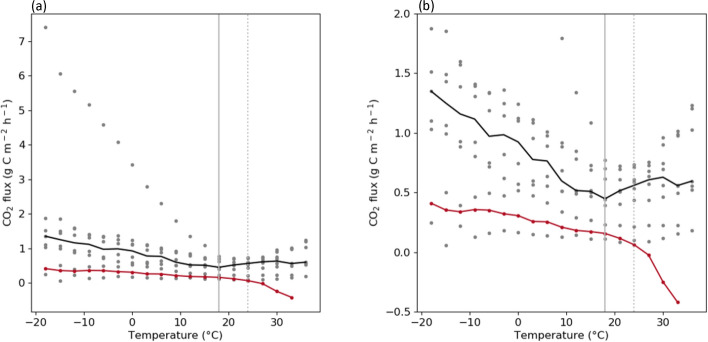
**a** Scatter plot of the hourly CO_2_ flux in accordance with the temperature for non-vegetation urban (gray) and vegetation (red) sites from 2017 to 2018. The black solid line represents the median value of the CO_2_ flux of all urban sites except for the vegetation site; the red solid line represents the CO_2_ flux of the vegetation site. The gray vertical lines represent 18 and 24 ℃ (solid and dashed, respectively), which are threshold temperatures for non-vegetated and vegetated sites, respectively. **b** The same as **a** but with a narrower y-axis range.

### Diurnal variations in each season

Diurnal patterns of the CO_2_ flux were similar in all seasons; however, notable differences existed between urban land-use types (Fig. 8). On average, the diurnal cycle of the CO_2_ flux in residential areas showed a distinct bimodal cycle, with maximum CO_2_ fluxes at 08:00 and 19:00 (local time) and minimum fluxes at 03:00 and 14:00 (Fig. 8a and d). In contrast, commercial areas had a relatively unimodal diurnal cycle, with a minimum at 04:00 and maximum at 12:00 (Fig. 8b and e). The vegetation area showed a diurnal variation with minimum and maximum CO_2_ fluxes at 13:00 and 00:00, respectively, in both summer and winter (Fig. 8c and f). However, the diurnal cycle was much clearer during the summer than that during winter. In the vegetation area, a negative CO_2_ flux value of − 0.36 C g m^− 2^ h^− 1^ occurred during the daytime (08:00–17:00), which was the only period with more CO_2_ absorption than emission.

To understand the causes of these diurnal variations in the CO_2_ flux, we compared them with the floating population and traffic volume data, which represented the major human activities (Fig. 9). In addition to human activities, the relationship between the CO_2_ flux and temperature was also examined to identify the meteorological effect. The CO_2_ flux in residential areas was negatively correlated with the floating population, while that in commercial areas was positively correlated with the floating population. This difference was attributed to the opposite diurnal cycle of the floating population in each area. In residential areas, the floating population was lower during the daytime than that at night; however, in commercial areas, the diurnal variation of the floating population showed the opposite trend (data not shown). Traffic volume and CO_2_ flux were positively correlated with a high average correlation coefficient of 0.8 in both residential and commercial areas. The CO_2_ flux and temperature were positively correlated in most non-vegetation sites. However, only the ILS vegetation site showed a negative correlation between the CO_2_ flux and temperature.

To understand variations in the correlation between the temperature and CO_2_ flux at each site, we compared the hourly CO_2_ flux with temperature, as shown in Fig. 10. The hourly temperature and CO_2_ flux presented different relationships based on the threshold of 18 ℃ in non-vegetated sites. In most non-vegetated urban sites, the CO_2_ flux increased as the temperature decreased below 18 ℃ (on average, by 0.026 C g m^− 2^ h^− 1^ ℃^−1^; p < 0.1), and it also increased as the temperature exceeded 18 ℃ (by 0.0074 g m^− 2^ h^− 1^ ℃^−1^; p < 0.1). Given that the threshold temperature of heating in Seoul is 18 ℃, as determined by the KMA, this result indicates that the CO_2_ flux data reflected the effect of the changing energy use owing to heating and cooling systems. For the vegetation site, the CO_2_ flux decreased as the temperature increased; however, the decreasing trend of the CO_2_ flux significantly increased once the temperature exceeded 24 ℃. The sensitivity of CO_2_ absorption to temperature increased from − 0.0075 to 0.056 C g m^− 2^ h^− 1^ ℃^−1^, a 7.44-fold increase compared with that of the previous temperature, as the temperature exceeded 24 ℃. This result is also consistent with that of a previous study, which showed that the amount of photosynthesis increases as the temperature increases to a certain level [54].

## Discussion

In this study, we analyzed CO_2_ flux observations at nine urban sites with different landscapes in the Seoul Capital Area for the first time. According to the results, the Seoul Capital Area showed substantially different CO_2_ flux levels, from 1.09 to 16.28 kg C m^− 2^ year^− 1^ among different land-use types. Previous studies showed various amounts of CO_2_ flux in different cities: 5.29 kg C m^− 2^ year^− 1^ in Heraklion, Greece; 12.72 kg C m^− 2^ year^− 1^ in London, England; 1.76 kg C m^− 2^ year^− 1^ in Helsinki, Finland; 4.90 kg C m^− 2^ year^− 1^ in Beijing, China; and 6.71 kg C m^− 2^ year^− 1^ in Vancouver, Canada [12, 13, 15, 20, 24]. Compared to those in previous studies, the regional differences in CO_2_ flux within the Seoul Capital Area were greater than those between cities. This indicates a considerable diversity in CO_2_ flux within cities.

Overall, in residential and commercial areas, where human activities are concentrated, CO_2_ emissions was five times higher than that in the vegetation and baseline areas. Among them, the total CO_2_ flux per year in SGN, an old town residential area comprising old buildings with low combustion efficiency, were over three times higher than those in other relatively new residential areas, JNG and GJW. Most CO_2_ emission from SGN occurred in the winter, reflecting the use of fossil fuels for winter heating. This indicates that heating-related CO_2_ can be greatly affected by the year of building construction, which is related to the combustion efficiency. Unlike non-vegetation urban areas, the ILS vegetation area absorbed CO_2_ during the daytime in summer. The average total amount of CO_2_ absorbed in the vegetation area during daytime (08:00–17:00) was 2.26 g C m^− 2^ d^− 1^. Considering that a highly vegetated suburban area of Baltimore, Maryland, USA, absorbs 1.25 g C m^− 2^ d^− 1^ of CO_2_ during daytime in the summer [55], we inferred that carbon uptake by vegetation actively occurs in ILS. However, this amount of CO_2_ absorbed during the daytime in summer is insufficient to eliminate the 3.37 g C m^− 2^ d^− 1^ of CO_2_ emissions at night from this region. This indicates that the amount of CO_2_ absorbed in urban vegetation areas is insufficient to balance the CO_2_ emissions from the city.

Previous studies that analyzed the cause of the variations in the CO_2_ flux in a city with limited observation sites showed that the CO_2_ flux in the city was mostly affected by the volume of traffic and vegetation area [12, 13, 15, 17, 20]. However, according to the results of this study obtained using multiple observation sites, the degree to which each driving factor (i.e., building energy usage, NDVI, floating population, traffic volume, and temperature) affects the CO_2_ flux variations varied for each urban land-use type. This result can only be obtained by using multiple observation sites. This also demonstrates that conducting CO_2_ flux observations in various locations across a city is necessary to determine the cause of variation in urban CO_2_ flux.

**Fig. 11:**
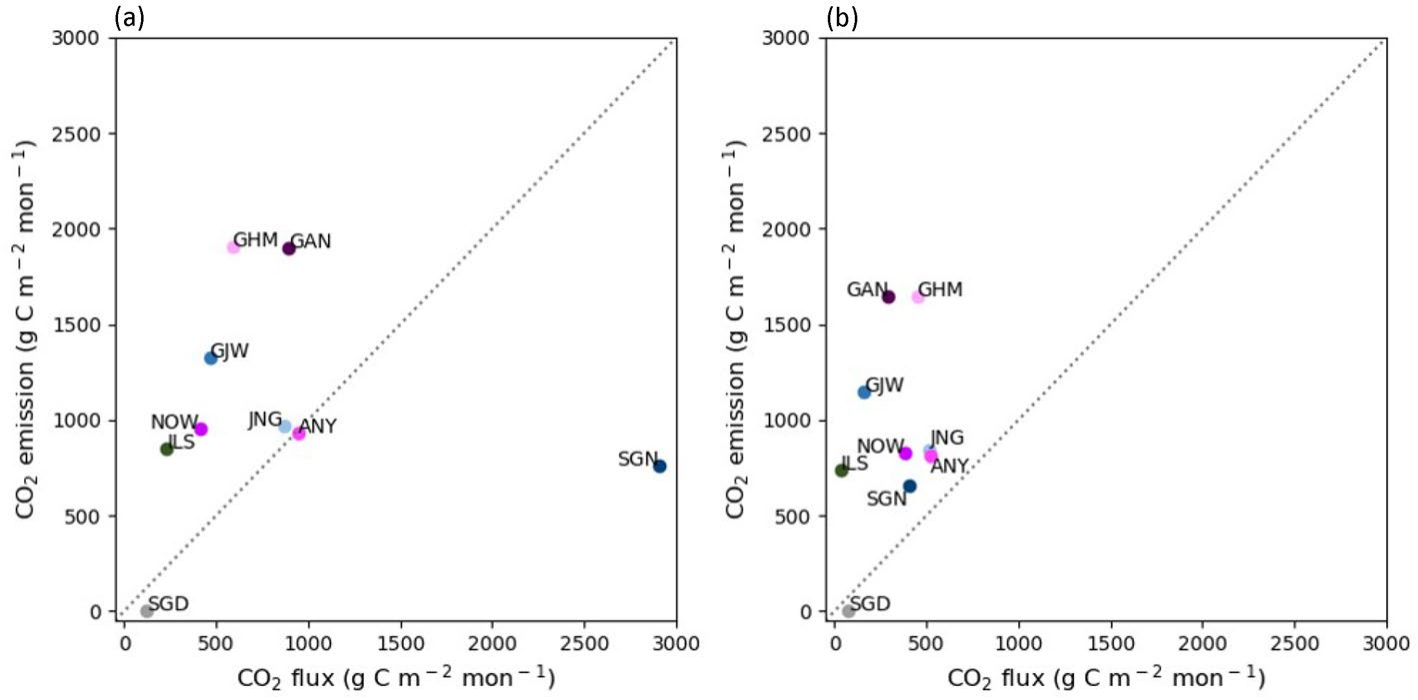
Scatter plot of monthly total observation-based CO_2_ flux and inventory-based CO_2_ emissions during **a** winter and **b** summer. The dotted line indicates a 1:1 line of CO_2_ flux and CO_2_ emissions

Expansion of the urban CO_2_ flux observation sites also enables cross-validation of inventory-based CO_2_ emissions. We compared the observation-based monthly CO_2_ flux data from this study with the inventory-based monthly CO_2_ emission data for each observation site (Fig. 11). This comparison was conducted for the winter (December, January, and February) and summer (June, July, and August) seasons, when vegetation had the least and greatest impact on observation-based CO_2_ flux data. The results of the winter season highlighted a substantial difference between the inventory-based CO_2_ emission and observation-based CO_2_ flux, with the exception of ANY, where there was only an 8.19 g C m^− 2^ mon^− 1^ difference between the two datasets (Fig. 11a). Aside from SGD and SGN, inventory-based CO_2_ emissions tended to be overestimated compared to observation-based CO_2_ flux; for example, the inventory-based CO_2_ emissions was 104.29 g C m^− 2^ mon^− 1^ greater at JNG, while it was 1316.54 g C m^− 2^ mon^− 1^ higher at GHM. By contrast, SGN was the only site where inventory-based CO_2_ emissions were an underestimate of observation-based CO_2_ flux. This may be because the high CO_2_ emissions due to the low combustion efficiency of SGN is not reflected in the inventory data. In the case of summer, on average, CO_2_ emission from the inventory data was overestimated by 694.40 g C m^− 2^ mon^− 1^ than the observation-based CO_2_ flux (Fig. 11b), which was more than twice larger than that of the winter (286.87 g C m^− 2^ mon^− 1^). Unlike inventory-based CO_2_ emission data, as the vegetation activity is reflected in the observation-based CO_2_ flux data, the difference between the two datasets could be widened in the summer when vegetation uptake is most active during the year. SGD has a 120.65 g C m^− 2^ mon^− 1^ and 71.36 g C m^− 2^ mon^− 1^ of observation-based CO_2_ flux for winter and summer seasons; however, the inventory determines its emissions to be zero as it is located on the coast, making it a difficult site to use for comparison.

These results indicate that variations in CO_2_ emission characteristics that may not be reflected in inventory data, may be identified through EC observations. This suggests that observation-based CO_2_ flux data may be used to evaluate actual CO_2_ emission characteristics at a specific location in real-time; however, EC observations cannot be conducted in all areas of the city. By contrast, inventory data is advantageous as it can consistently quantify and manage CO_2_ emissions in all regions. This means more accurate CO_2_ emission characteristics of an urban area may be reproduced by narrowing the gap between inventory-based CO_2_ emissions and observation-based CO_2_ flux through cross-validation.

The vegetation and non-vegetation urban areas showed different relationships between the hourly CO_2_ flux and temperature. In the vegetation area, the decreasing trend of CO_2_ flux significantly increased at 24 ℃, indicating that CO_2_ absorption from photosynthesis increased rapidly after the temperature increased above a certain level. Non-vegetation urban areas showed the opposite correlation between the CO_2_ flux and temperature based on the 18 ℃ threshold. This result indicates that CO_2_ emissions have different sensitivities to cold and hot temperatures, being over three times more sensitive to cold temperatures. In Seoul, 18 ℃ is the threshold temperature for indoor heating, suggesting that the relationship between the CO_2_ flux and temperature is related to the energy use from building heating and cooling. In fact, the gas usage of buildings in non-vegetated urban sites peaked in the summer and winter, when the temperature was the highest and lowest of the year, respectively. Furthermore, this result is consistent with the v-shaped temperature dependence pattern of energy demand, which is high at low and high temperatures [56]. Based on the threshold temperature for heating and cooling demands (Tb), a lower temperature indicates heating demand, and a higher temperature indicates cooling demand. The energy demand at Tb is the amount of energy that is used consistently regardless of temperature. Applying this energy demand concept to our results showed that CO_2_ emissions in the Seoul Capital Area were more sensitive to the heating demand than to the cooling demand. Additionally, an average of 0.45 g C m^− 2^ h^− 1^ of CO_2_ was emitted from the Seoul Capital Area from the use of fixed energy, regardless of the temperature.

## Conclusions

Global warming has caused extreme variabilities in weather conditions, including unprecedented heat and cold waves [57, 58]. Based on the relationship between temperature and CO_2_ emissions from our study, CO_2_ emissions from urban areas are expected to increase as extreme weather conditions become more frequent. In particular, the sensitivity of CO_2_ emissions to temperature fluctuations from global warming will be the greatest in places such as the old town residential area, where CO_2_ emissions from heating are more than three times greater than those in other areas. However, this also implies that if urban planning is implemented to improve the energy efficiency of the old downtown area, CO_2_ emissions from the city can be effectively reduced. As climate change intensifies, the management of old downtown areas with low energy efficiency should be prioritized.

This study demonstrates that to identify the comprehensive CO_2_ flux characteristics of cities, observations are required for each area to reflect the complexities of urban land-use types. As a novel study, we improved the understanding of the CO_2_ flux characteristics according to urban land-use types using multiple CO_2_ flux observations from various urban landscapes. The CO_2_ flux characteristics derived in this study can help policymakers to develop effective CO_2_ reduction policies, thereby enabling cities to play important roles in mitigating climate change.

**Table 1:** CO_2_ flux observations

Site code	Full name	Lat. (°N)	Long. (°E)	Classification	Land cover (%)	Annual CO_2_ flux (kg C m^− 2^ year^− 1^)	
						2017	2018
JNG	Jungnang	127.079	37.591	Residential	Road (36), Residence (29), Commercial, industrial, and public facilities (18), Urban vegetation (8)	6.90	7.85
GJW	Gajwa	126.914	37.584	Residential	Road (39), Residence (28), Commercial, industrial, and public facilities (12), Urban vegetation (12)	3.27	3.19
SGN	Seongnam	127.142	37.441	Residential (old town)	Road (34), Residence (30), Commercial, industrial, and public facilities (23), Urban vegetation (9)	16.49	16.06
GHM	Gwanghwamun	126.978	37.572	Commercial	Road (45), Commercial, industrial, and public facilities (30), Urban vegetation (12), Residence (4)	5.60	5.30
ANY	Anyang	126.960	37.394	Commercial	Road (50), Urban vegetation (21), Commercial, industrial, and public facilities (18), Residence (5)	8.33	6.60
NOW	Nowon	127.056	37.654	Commercial	Road (49), Urban vegetation (19), Commercial, industrial, and public facilities (14), Residence (7)	4.72	4.46
GAN	Gangnam	127.022	37.520	Commercial	Road (42), Commercial, industrial, and public facilities (24), Urban vegetation (15), Residence (13)	5.83	6.25
ILS	Ilsan	126.731	37.685	Vegetation		1.66	1.63
SGD	Songdo	126.655	37.383	Baseline	Road (48), Urban vegetation (32), Commercial, industrial, and public facilities (11), Residence (3)	1.15	1.02

## Data Availability

The datasets of the land cover map, GIS building integrated information, NDVI, ODIAC, building energy usage, traffic volume, and floating population supporting the conclusions of this article are available at: https://egis.me.go.kr/, http://www.nsdi.go.kr/, https://earthdata.nasa.gov/, https://db.cger.nies.go.jp/dataset/ODIAC/, https://open.eais.go.kr/, http://topis.seoul.go.kr/, and https://data.seoul.go.kr/, respectively. CO_2_ flux and temperature data from the eddy covariance observation system are available from the data developers upon request.

## Abbreviations

CO_2_: Carbon dioxide
EC: Eddy covariance
KMA: Korea Meteorological Administration
SGD: Songdo
JNG: Jungnang
GJW: Gajwa
SGN: Seongnam
GHM: Gwanghwamun
ANY: Anyang
NOW: Nowon
GAN: Gangnam
ILS: Ilsan
NIMS: National Institute of Meteorological Sciences
NDVI: Normalized Difference Vegetation Index
ODIAC: Open-Data Inventory for Anthropogenic Carbon dioxide
NIES: National Institute for Environmental Studies, Japan
MODIS: Moderate Resolution Imaging Spectroradiometer
T_b_: threshold temperature between heating demand and cooling demand
